# Effects of Wee1 inhibitor adavosertib on patient-derived high-grade serous ovarian cancer cells are multiple and independent of homologous recombination status

**DOI:** 10.3389/fonc.2022.954430

**Published:** 2022-08-23

**Authors:** Pia Roering, Arafat Siddiqui, Vanina D. Heuser, Swapnil Potdar, Piia Mikkonen, Jaana Oikkonen, Yilin Li, Sanna Pikkusaari, Krister Wennerberg, Johanna Hynninen, Seija Grenman, Kaisa Huhtinen, Annika Auranen, Olli Carpén, Katja Kaipio

**Affiliations:** ^1^ Institute of Biomedicine and Finnish Cancer Center (FICAN) West Cancer Centre, University of Turku and Turku University Hospital, Turku, Finland; ^2^ High Throughput Biomedicine Unit, Institute for Molecular Medicine Finland (FIMM), University of Helsinki, Helsinki, Finland; ^3^ Helsinki Institute of Life Science (HiLIFE), Institute for Molecular Medicine Finland (FIMM), University of Helsinki, Helsinki, Finland; ^4^ Research Program in Systems Oncology, University of Helsinki and Helsinki University Hospital, Helsinki, Finland; ^5^ Biotech Research and Innovation Centre (BRIC), University of Copenhagen, Copenhagen, Denmark; ^6^ Department of Obstetrics and Gynecology, Turku University Hospital and University of Turku, Turku, Finland; ^7^ Department of Obstetrics and Gynecology and Tays Cancer Centre, Tampere University Hospital, Tampere, Finland; ^8^ Department of Pathology, Precision Cancer Pathology, University of Helsinki and Helsinki University Hospital, Helsinki, Finland

**Keywords:** adavosertib, patient-derived cell line, high-grade serous ovarian cancer (HGSOC), homologous recombination (HR), drug screen, platinum-resistant ovarian cancer, homologous recombination deficiency (HRD), homologous recombination proficient

## Abstract

**Objective:**

A major challenge in the treatment of platinum-resistant high-grade serous ovarian cancer (HGSOC) is lack of effective therapies. Much of ongoing research on drug candidates relies on HGSOC cell lines that are poorly documented. The goal of this study was to screen for effective, state-of-the-art drug candidates using primary HGSOC cells. In addition, our aim was to dissect the inhibitory activities of Wee1 inhibitor adavosertib on primary and conventional HGSOC cell lines.

**Methods:**

A comprehensive drug sensitivity and resistance testing (DSRT) on 306 drug compounds was performed on three patient-derived genetically unique HGSOC cell lines and two commonly used ovarian cancer cell lines. The effect of adavosertib on the cell lines was tested in several assays, including cell-cycle analysis, apoptosis induction, proliferation, wound healing, DNA damage, and effect on nuclear integrity.

**Results:**

Several compounds exerted cytotoxic activity toward all cell lines, when tested in both adherent and spheroid conditions. In further cytotoxicity tests, adavosertib exerted the most consistent cytotoxic activity. Adavosertib affected cell-cycle control in patient-derived and conventional HGSOC cells, inducing G2/M accumulation and reducing cyclin B1 levels. It induced apoptosis and inhibited proliferation and migration in all cell lines. Furthermore, the DNA damage marker γH2AX and the number of abnormal cell nuclei were clearly increased following adavosertib treatment. Based on the homologous recombination (HR) signature and functional HR assays of the cell lines, the effects of adavosertib were independent of the cells' HR status.

**Conclusion:**

Our study indicates that Wee1 inhibitor adavosertib affects several critical functions related to proliferation, cell cycle and division, apoptosis, and invasion. Importantly, the effects are consistent in all tested cell lines, including primary HGSOC cells, and independent of the HR status of the cells. Wee1 inhibition may thus provide treatment opportunities especially for patients, whose cancer has acquired resistance to platinum-based chemotherapy or PARP inhibitors.

## Introduction

There is an urgent need for effective therapies for patients with platinum-resistant high-grade serous ovarian cancer (HGSOC). While most HGSOC patients initially respond to the standard first-line platinum–taxane combination chemotherapy, relapse within 18 months is common followed by chemoresistance (1, 2). Reasons for relapse and treatment failure vary, and the progress in improving clinical care has been rather slow. The heterogeneity as well as adaptability of the HGSOC genome to chemotherapy requires new approaches to improve the outcome of the disease (3). A molecular indicator of platinum sensitivity is homologous recombination deficiency (HRD), through either genetic or epigenetic alterations (4). PARP inhibitors have been recently shown to provide a significant clinical benefit for HGSOC patients, but they are effective only on HRD tumors (5, 6). Therefore, there is a special need to identify compounds that are efficient regardless of the homologous recombination status.

*In vitro* models are essential for identifying effective oncology compounds or drug combinations for any type of cancer, including HGSOC. Until recently, most of the *in vitro* studies have been conducted using publicly available ovarian cancer cell lines that may not represent the HGSOC subtype and that may have undergone a variety of *in vitro* alterations during extensive passaging (7). To overcome this potential hurdle, we created patient-derived HGSOC cell lines and demonstrated that the cells can be cultured and tested under conditions that mimic their stemness properties (8). The cell lines can provide a valuable model of the heterogenous disease and identify personalized treatment options.

Here we tested a panel of 306 drug compounds on HGSOC cells and identified potential effective compounds for further studies (9, 10). Altogether, three patient-derived and two conventional HGSOC cell lines, containing both HRD and HR proficient (HRP) cell types, were screened using the panel, to reveal interesting pharmacologically active substances for further investigation.

In this study, we have focused on drug candidates that have shown effectiveness in most cell models and under both traditional and stemness-like growth conditions. Our rationale was that the selected compounds had the potential for a broader and fast clinical translation. Another criterion was that the candidates must and have been included in clinical trials with any type of cancer and shown promising results in early studies. Of the tested compounds, the Wee1 inhibitor adavosertib (AZD1775) showed the most consistent inhibitory results. Wee1 kinase plays a crucial role in cell-cycle regulation and DNA damage identification and repair in malignant and non-malignant cells, and its inhibition has shown promising results in early phase clinical trials (11–14), including HGSOC. Therefore, we carried out a more detailed analysis on its effects on all five cell lines.

## Material and methods

### Patients

Tumor and ascites material and clinical information was collected from consenting patients treated at the Department of Obstetrics and Gynecology, Turku University Hospital, Turku, Finland, as described previously (8, 15). The patients participated in a clinical trial (NCT01276574) and were diagnosed with stage III or IV HGSOC, verified by histopathological evaluation and imaging. Treatment-naive ascites was collected during diagnostic laparoscopy. Patients who were considered primarily inoperable received three cycles of neoadjuvant chemotherapy (NACT), and new samples were taken during the interval debulking surgery (IDS). For this study, we used cell lines from three patients (OC002, M022i, and M048i). The patients' age range at the time of the diagnosis was between 61 and 66 years. Progression-free survival (PFS) was 3.1 to 10.1 months and overall survival (OS) 4.0 to 35.8 months. Detailed clinical information is presented in **Table S1**.

### Cell culture

Two patient-derived cell lines were established from ascites (OC002 and M022i), of which OC002 was treatment-naive and M022i was from IDS. One cell line originated from omental metastasis (M048i) and was from IDS (8). The cell lines were characterized by DNA sequencing (see *DNA/RNA sequencing and functional assessment of homologous recombination capacity*). In addition to these patient-derived cell lines, two conventional HGSOC cell lines were explored: CAOV3 (RRID:CVCL_0201, American Type Culture Collection, ATCC, USA) and OVCAR8 (RRID:CVCL_1629, National Cancer Institute, NCI, USA).

Cells were grown at 37°C, at 5% CO_2_. The OVCAR8 cell line was cultured in an RPMI medium; OC002, M022i, M048i, and CAOV3 cells were cultured in a DMEM-F12-based spheroid medium as described previously (8). To sustain adherent cell cultures for IncuCyte experiments and immunostainings, a modified OCMI medium was used instead of a spheroid medium: 1:1 of medium 199 (Gibco) and DMEM/F-12 (Lonza) supplemented with 5% FBS (Lonza), 2% ITS (Corning), 100 µg/ml penicillin/streptomycin (Gibco Life Technologies), 0.5 ng/ml 17 beta-estradiol (Merck), 0.2 pg/ml triiodothyronine (Sigma), 0.025 µg/ml all-trans retinoic acid (Merck), 13.75 µg/ml insulin (Sigma), 25 ng/ml cholera toxin (Sigma), 0.5 µg/ml hydrocortisone (Sigma), and 10 ng/ml EGF (Gibco Life Technologies).

### DNA/RNA sequencing and functional assessment of homologous recombination capacity

To genetically characterize tumors and identify patient-specific *TP53* mutations, we sequenced available fresh frozen tissue or ascites samples, whole-blood buffy coat samples (germline reference), and/or cells from the cultures. Germline reference was available for M022i and M048i, fresh frozen tumor tissue or ascites for M048i (N = 4) and OC002 (N = 1), and cultured cells for M022i and M048i. DNA/RNA was extracted from samples with AllPrep DNA/RNA Mini Kit (Qiagen). Sequencing was performed in the BGI (Beijing Genomics Institute) as whole-genome sequencing (WGS) with HiSeq X Ten or with whole exome sequencing (WES) with Agilent SureSelect Human All Exon V5 using HiSeq 2000.

Data were aligned to GRCh38.d1.vd1 (median coverage 48, **Table S2**), and mutations were called with Mutect2 [GATK4 (16)]. Mutation pathogenicity was evaluated using COSMIC (17), ClinVar (18), and CADD (19) for exonic non-synonymous mutations, indels, and splicing. Cell identity was confirmed with contamination test (GATK4) for M022i and M048i where sequencing data from both cells and germline reference were available. All patients were identified with high variant allele frequency, pathogenic *TP53* mutations, which were used to verify cell identity in the cultures (**Table S2**). In addition, the mutational status of other HR-related genes was identified (list of genes in **Table S3**). Mutational signatures were fitted with COSMIC v3.1 SBS signatures based on SigProfiler attribution (20) to assess HRD mutational signature SBS3.

Functional homologous recombination status was analyzed for OC002 and M048i cells as described in (21). Epithelial cells (cytokeratin positive) in the G2 phase (cyclin A2 positive) were stained with RAD51 to distinguish between RAD51-positive and -negative cells. HR-score was obtained by calculating the percentage of RAD51-positive cells. At least 300 cells were counted per sample. HR-scores below 35% were considered HRD (**Table S2**).

To investigate whether the cell lines were different regarding multidrug resistance (MDR), we analyzed RNA expression data of the ABC transporters (ATP-binding cassette transporters). The results are provided as reads per kilobase per million (RPKM) values, normalized to GAPDH expression. A heatmap was produced using Log2+1 values at the publicly available web software Heatmapper (heatmapper.ca) (22).

### High-throughput drug sensitivity and resistance testing

The five cell lines were subjected to high-throughput screening (HTS) with a panel of 306 clinical FDA- and/or EMA-approved and emerging oncology drug compounds (**Table S4**). Screening was performed at the Institute for Molecular Medicine Finland (FIMM) as described previously (9, 10). While a part of the drug screen data has been published earlier (8), here two additional patient-derived HGSOC cell lines are presented: M048i and OC002. Cells were tested in five different drug concentrations spanning a relevant 10,000-fold concentration range for each individual drug in conventional adherent and stem-like spheroidal cell culture conditions as was described earlier (8). Cell viability was measured with CellTiter-Glo (Promega, Madison, Wisconsin, USA) after 72 h of exposure to the drugs in at least five separate experiments with triplicate wells.

The data were analyzed with the quantitative scoring approach, where a multiparameter area under a curve sensitivity calculation called the drug sensitivity score (DSS) was used (9, 10). This integration combines the model-based and area-based drug response calculations. The DSS was calculated for each drug, and responses were compared to human healthy bone marrow-averaged controls to evaluate the specific selective DSS (sDSS) as previously described (9, 10). Previously reported cutoff values of sDSS were used: sDSS >5 for effective drugs and sDSS >10 for highly effective drugs (23). The drug sensitivity and resistance testing (DSRT) data were analyzed using the web-based pipeline BREEZE (https://breeze.fimm.fi/) (24).

The cytotoxic effect of adavosertib (Selleckchem, Munich, Germany) was further validated with a CellTiter-Glo^®^ (Promega) cell viability test. To test cells' sensitivity to cisplatin, 5,000 cells/well were plated on a 96-well plate in triplicates. Cisplatin was added in concentrations of 0.01–100 µM, and cell viability was measured after 72 h of incubation. Luminescence was detected with a Victor2 luminometer (Wallac, Turku, Finland). The IC50 value of adavosertib for each cell culture was calculated, and a dose of 500 nM was selected to be used for the functional experiments. The IC50 value for cisplatin was calculated using log-transformed data and logarithmic trend line.

### Flow cytometry

The M048i, OC002, CAOV3, and OVCAR8 cells were investigated with a BD LSRFortessa™ flow cytometer (BD Biosciences, NJ, USA). For each measurement, 10,000–30,000 events were assessed. The flow cytometry data were analyzed with Flowing Software 2.5.1 (Mr. Perttu Terho, Turku Bioscience Centre, Turku, Finland). Duplicates of each sample were tested, and the experiment was repeated a minimum of three times.

Early and late apoptoses were detected after 48 and 72 h of treatment with adavosertib with an Annexin V-FITC Apoptosis Detection Kit (ab14085, Abcam). Samples were processed according to the kit's protocol.

Cell-cycle phases were detected with a Click-iT EdU Flow Cytometry Assay Kit Pacific Blue (C10425, Invitrogen). Cell-cycle progression was studied in vehicle- and adavosertib-treated cells at time points 24, 48, and 72 h. Samples were collected, and the protocol was performed according to the manufacturers' instructions.

### Cell proliferation

Cell proliferation of the HGSOC cell lines was inspected for 72 h at 2-h intervals with an IncuCyte S3 high-content imager (Essen BioScience, Ann Arbor, MI). Cells were plated in 96-well plates (Greiner Bio-One) to be 10% confluent and treated with 500 nM adavosertib. Cells treated with vehicle (DMSO) were used as control in all the experiments. Each sample was measured in triplicate, and the experiments were repeated a minimum of three times. Proliferation was measured by confluence area by IncuCyte software (Essen BioScience).

### Wound healing assay

Wound healing of the HGSOC cells was measured for 72 h at 2-h intervals with an IncuCyte S3 high-content imager (Essen Bioscience, Ann Arbor, MI). Experiments were performed on 96-well plates (ImageLock, Essen BioScience) with adavosertib (500 nM) or vehicle (DMSO). Each sample was measured in triplicate, and the experiments were repeated a minimum of three times. Relative wound density was analyzed by IncuCyte software (Essen BioScience). The wells were precoated with Geltrex (Gibco) for migration experiments and with Matrigel (100 µg/ml, Corning, Bedford, MA, USA) for invasion experiments. Wells were pretreated for 24 h with adavosertib (500 nM) before wound making with a wound-maker provided with IncuCyte S3 (Essen Bioscience). In the invasion experiments after wound making, the cells were covered with 50 µl Matrigel (2 mg/ml) for 30 min in the incubator; thereafter, adavosertib was added.

### Western blotting

The effect of adavosertib on the cell cycle was investigated in the cell cultures treated for 72 h. Cells were grown in a DMEM/F-12 or RPMI medium and harvested and lysed with a RIPA Buffer supplemented with protease inhibitors. Protein concentrations were measured using a Bio-Rad protein assay kit according to the manufacturer's instructions. Equal amounts of proteins in the Laemmli buffer were separated in 4%–20% polyacrylamide PROTEAN^®^ TGX™ Precast Protein Gels (Bio-Rad) and transferred to the 0.2-µm PVDF membrane using the Trans-Blot Turbo Transfer System (Bio-Rad). Membranes were blocked with 5% BSA (bovine serum albumin) in Tris-buffered saline with 0.05% Tween 20 (TBST) and probed with primary antibodies diluted in the same solution. Primary antibodies used in the Western blotting were rabbit monoclonal anti-Cyclin B1 (D5C10, 1:1,000, Cell Signaling), rabbit monoclonal anti-Cyclin E1 (EP435E, 1:500, Abcam), mouse anti-PCNA (PC10, 1:2,000, Cell Signaling), and mouse monoclonal anti-γH2AX (phospho S139, 1:1,000, Abcam). GAPDH-HRP-conjugated (1:5,000, Abcam) or mouse monoclonal anti-α-Tubulin (B-5-1-2, 1:1,000, Sigma) was used as control for protein loading. The secondary antibodies were HRP-conjugated swine anti-rabbit and rabbit anti-mouse immunoglobulins (1:2,500, Dako, Glostrup, Denmark) diluted in a blocking solution. Membranes were washed three times with TBST between antibody incubations. Bound proteins were detected by enhanced chemiluminescence using ChemiDoc™ Gel Imaging System (Bio-Rad), and the signals were quantified using ImageJ.

### Immunostaining and nuclear morphology analysis

Cells were grown on glass slides with a Geltrex (Gibco) coating and a modified OCMI or RPMI medium. After 72 h of incubation with 500 nM adavosertib, the cells were fixed for 10 min in 4% paraformaldehyde and washed with PBS. Blocking was performed with 5% BSA and 0.5% Triton X-100 in PBS for 30 min. Slides were incubated at room temperature for 60 min with a primary antibody diluted in a blocking buffer and thereafter for 60 min with a secondary antibody. The slides were washed twice after the antibody incubations with PBS and embedded in a mounting medium containing DAPI for staining nuclei (ProLong Gold Antifade Mountant with DAPI, Thermo Fisher). The following primary antibodies were diluted in the blocking buffer: mouse monoclonal anti-α-Tubulin (B-5-1-2, 1:100, Sigma) and mouse monoclonal anti-γH2AX (phospho S139, 1:500, Abcam). Alexa Fluor 555 Donkey anti-Mouse (1:400, Invitrogen) was used as a secondary antibody together with Alexa Fluor 488-conjugated phalloidin (1:300, Invitrogen, Carlsbad, CA) for actin filament visualization.

Nuclear morphology was assessed after 72 h after treatment with vehicle or 500 nM adavosertib by staining the tubulin for cell structure and embedding in mounting medium containing DAPI for the cell nucleus staining. At least 100 cells were counted in each sample, and the nuclei were categorized as normal, abnormal, or multinuclear. Images were taken with a Nikon Eclipse Ni fluorescence microscope, and different channels were merged using ImageJ v1.53a software (http://rsbweb.nih.gov/ij/).

### Statistics

The IC50 values for the adavosertib cytotoxicity validation were acquired using a sigmoidal dose–response curve. The differences in proliferation, wound healing, cell cycle, apoptosis, nuclear abnormalities frequencies, and protein levels between vehicle- and adavosertib-treated cells were compared using the two-sided *t*-test on freely available *VassarStats: Website for Statistical Computation* (www.vassarstats.net). *P* values ≤0.05 were considered statistically significant.

## Results

### Several compounds effectively kill HGSOC cells

**Figure 1:**
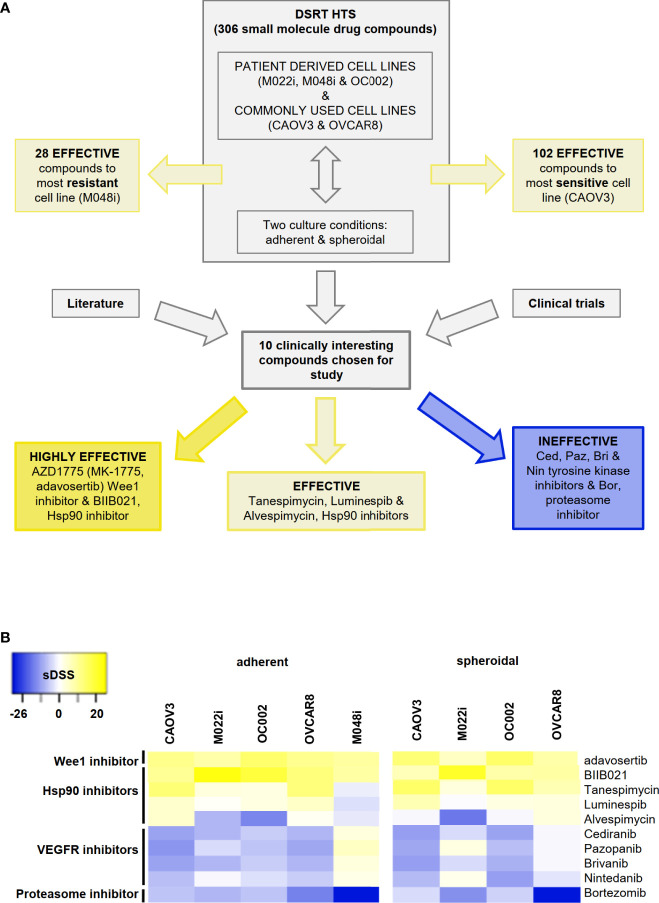
High-throughput drug sensitivity and resistance testing (DSRT) with 306 drug compounds. **(A)** Study design and basis for selection of small molecules for further analyses. DSRT was performed with five HGSOC cell lines in adherent and spheroidal culture conditions. The number of effective compounds varied between 28 (M048i) and 102 (CAOV3). Based on literature search and ongoing clinical trials, 10 compounds were selected for further cytotoxicity tests, which divided compounds into three categories: highly effective, effective, and ineffective. **(B)** Heatmap of the selected drug compounds based on sDSS (selective drug sensitivity score) values tested in adherent and spheroidal conditions. Bright yellow = sensitive, dark blue = resistant; sDSS >5 effective, sDSS ≥10 highly effective. M048i spheroidal data not available.

The drug sensitivity testing was performed under two growth conditions, i.e., conventional adherent and spheroid conditions inducing stemness features and mimicking the environment of the malignant cells in ascitic fluid. We employed a correlation plot analysis for sDSS to assess the condition-related variations in drug sensitivity (**Figure S5**). The number of effective (sDSS >5) drugs from the whole screen of 306 drug compounds varied between screened cell lines and was partially dependent on growth conditions. Of the primary HGSOC cell lines, M048i was sensitive to 28 compounds in adherent cell culture conditions, OC002 to 59 compounds, and M022i to 72 compounds. Of the conventional cell lines, OVCAR8 showed sensitivity to 74 compounds and CAOV3 to 102 compounds (**Figure 1A** and **Table S6**). The results demonstrate that compounds target cancer cells from individual patients differently, possibly reflecting the variability of individual patients' response to chemotherapy/targeted therapy. While there was variation between the patient-derived cell lines, they were generally more resistant than the conventional HGSOC cell lines.

**Figure 2:**
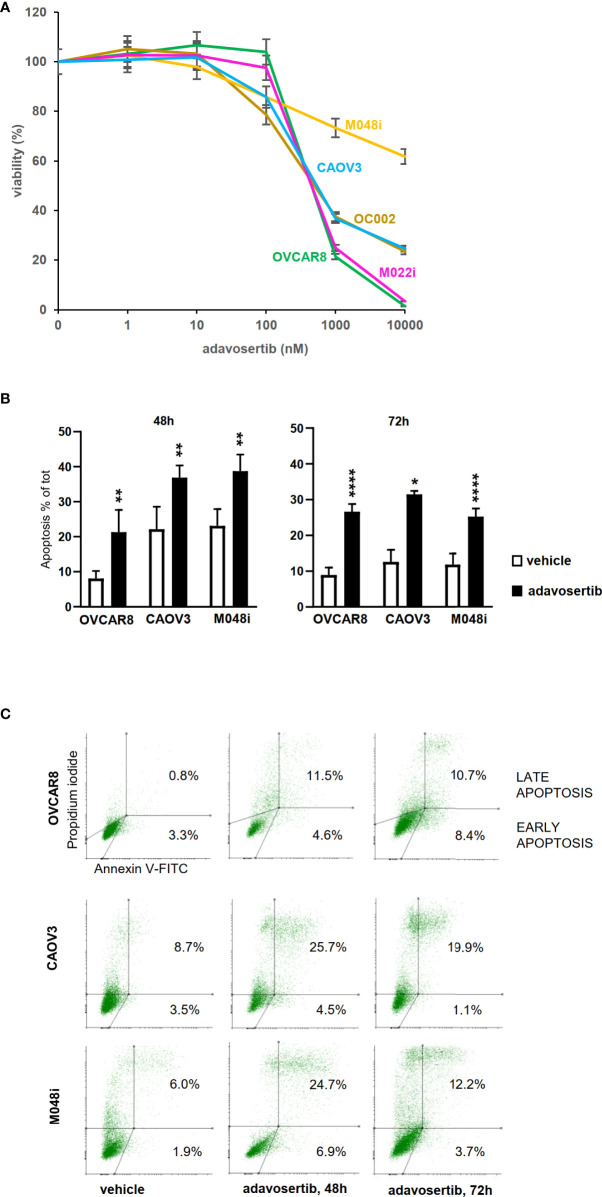
Effects of adavosertib on cell viability and apoptosis. **(A)** Viability was assessed after a 72-h treatment with adavosertib in five different concentrations (1–10,000 nM) (±SD). **(B)** Apoptosis was measured in three HGSOC cell lines (OVCAR8, CAOV3, and M048i) with annexin V staining detected by flow cytometry. Apoptotic cell populations (% of total cell amount, ±SD) were significantly elevated in all cell lines after 48 and 72 h of treatment with 500 nM adavosertib as compared with the control cells (p-value: ns = p > 0.05, *p ≤ 0.05, **p ≤ 0.01, and ****p ≤ 0.0001). **(C)** Distribution of early and late apoptotic cell populations (% of total number of cells).

Ten clinically interesting drug compounds from the initial screen were selected for further investigation according to two criteria: literature search and ongoing clinical studies in HGSOC or other cancers (**Figure 1A**). In more detailed cytotoxicity assays, the cellular responses to these 10 drug compounds were variable (**Figure 1B**). The patient-derived M048i cells were relatively resistant to all of the 10 oncology compounds. Similarly, M048i cells were extremely resistant to cisplatin (IC50 >100 µM) while in other cell lines IC50 varied between 1.3 and 11.7 µM (**Figure S7** and **Table S8**). Five of the drug compounds were categorized as highly effective (sDSS ≥10) in the majority of the HGSOC lines: Wee1 inhibitor adavosertib and four Hsp90 (heat shock protein 90) inhibitors BIIB021, tanespimycin, luminespib, and alvespimycin (**Figure 1B**). Of these compounds, the Wee1 inhibitor adavosertib was chosen for further research due to its ongoing clinical interest and the validation results. **Table S9** shows the sDSS values for the 10 investigated compounds tested in adherent or spheroid culture conditions. Adavosertib was cytotoxic for all cells except M048i, with IC50s between 578 and 785 nM (**Figure 2A** and **Table S9**).

### Adavosertib induces apoptosis and causes G2/M arrest in HGSOC cells

To study whether adavosertib induces apoptosis, HGSOC cells were treated for 48–72 h before labeling with Annexin V. Adavosertib-induced apoptosis was evident in all of the examined cell lines, surprisingly also including M048i, the most resistant cell line in viability tests (**Figures 2B, C**, **Table S10**). The total number of apoptotic cells after a 72-h treatment with adavosertib (500 nM) increased from 8.9% to 26.7% in OVCAR8, from 12.6% to 31.5% in CAOV3, and from 11.9% to 25.2% in the M048i cell line.

**Figure 3:**
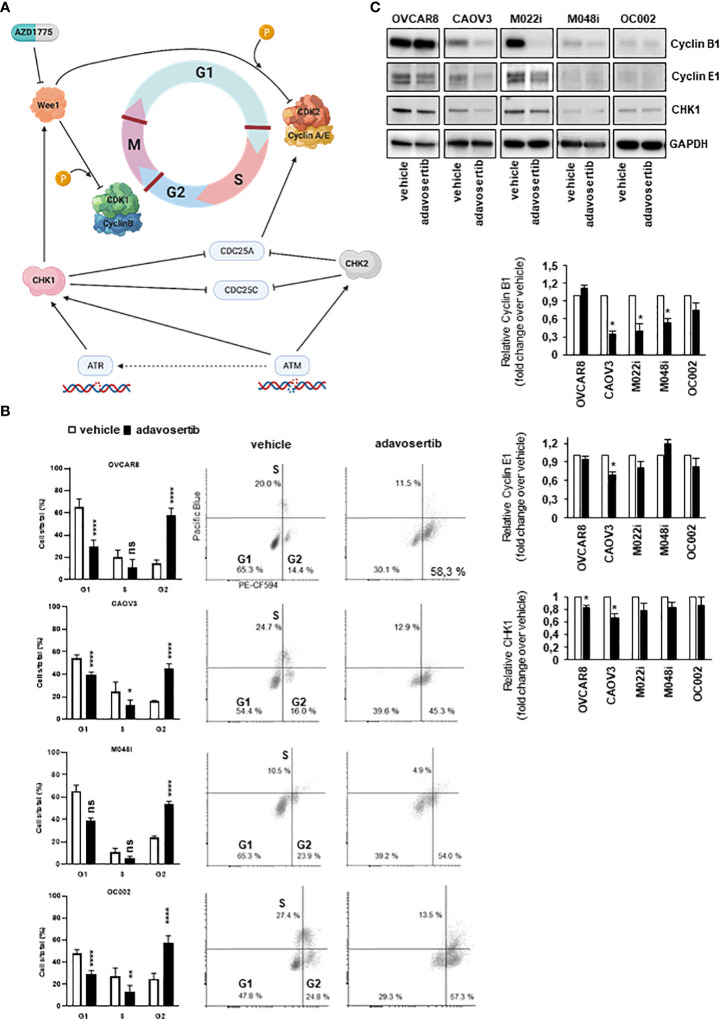
Effect of adavosertib on the cell cycle. **(A)** Illustration of cell-cycle checkpoints involved in DNA-damage response (DDR) pathways, including the role of Wee1. In HGSOC cells, the G1/S checkpoint is dysfunctional due to p53 mutation, while the G2/M checkpoint remains functional in DNA-damage repair. Inhibition of Wee1 by adavosertib (AZD1775) disables the G2/M checkpoint, thereby enabling a cell with damaged DNA to enter mitosis. Created with BioRender.com. **(B)** HGSOC cells were labeled with 5-ethynyl-2-deoxyuridine (EdU), and cell-cycle phases were monitored with flow cytometry. The distribution of cells in the G1, S, and G2/M phases is shown after 72 h of treatment with adavosertib (500 nM) or vehicle. White bars = vehicle and black bars = adavosertib treated (average, ±SD). Dot blots present the distribution of cells with EdU staining. **(C)** Western blot analysis of cyclin B1, cyclin E1, and Wee1 pathway regulating CHK1 protein in adavosertib- (500 nM) and vehicle-treated cells after 72 **(h)** The bars show quantitative analysis of cyclin B1, cyclin E1, and CHK1, normalized to GAPDH, using ImageJ software. The values shown are the mean ± SE of three separate experiments. Significant difference between vehicle and adavosertib treatment was determined by the *t*-test at ns = p > 0.05, *p ≤ 0.05, **p ≤ 0.01 and ****p ≤ 0.0001.

The effect of adavosertib on the cell cycle was examined after treatment for 24, 48, and 72 h. When compared with control cells, significant G2/M accumulation and a reduction in the G1 cell-cycle phase were found in all four evaluated cell lines (**Figure 3B**). As compared to vehicle-treated cells, adavosertib diminished the proportion of G1 cells by 27%–54%, dependent on the cell line. The accumulation of G2 cells was clear in all tested cell lines. In OVCAR8, the percentage of G2 cells of total cell amount after adavosertib treatment was 58.3% as compared to 14.4% in untreated cells. In CAOV3, M048i, and OC002 cell lines, the percentages of G2 cells in treated cells and vehicle were 45.3% and 16.0%; 54.0% and 23.9%; and 57.2% and 24.8%, respectively. In addition, a trend toward a reduced S phase was seen in all cell lines, but the difference was statistically significant only in CAOV3 and OC002 (**Figure 3B**, **Table S11**).

Interestingly, the cell-cycle regulation of the most resistant cell line M048i appeared abnormal. In the untreated samples, very few cells were in the S phase and the same trend was observed in the adavosertib-treated cells (**Figure 3B**). However, a drift from G1 to G2/M in the treated cells was detected, as expected. Although the result of the cell viability test showed relative resistance of M048i cells to adavosertib (**Figure 2A**), the increased apoptosis and G2/M accumulation suggest that adavosertib has some beneficial effect on these patient-derived HGSOC cells.

The cell-cycle alterations were further explored by Western blot analysis of cyclin E1 and cyclin B1, which regulate the G1/S and G2/M transition, respectively (**Figure 3A**). The level of cyclin B1 was reduced in most cell lines after adavosertib treatment, although it remained normal in OVCAR8 cells (**Figure 3C**). Cyclin E1 expression was reduced in CAOV3 and M022i following 72 h of adavosertib treatment (**Figure 3C**). Similarly, the CHK1 protein, which is a key regulator upstream of the Wee1 pathway (**Figure 3A**), was studied. After adavosertib therapy, there was a reduction in CHK1 expression (**Figure 3C**) in all cell lines. It is worth noting that the amounts of both cyclins and CHK1 differed greatly among the studied cell types, with M048i and OC002 expressing very low levels (**Figure 3C**).

### Adavosertib reduces proliferation and migration in HGSOC cells

**Figure 4:**
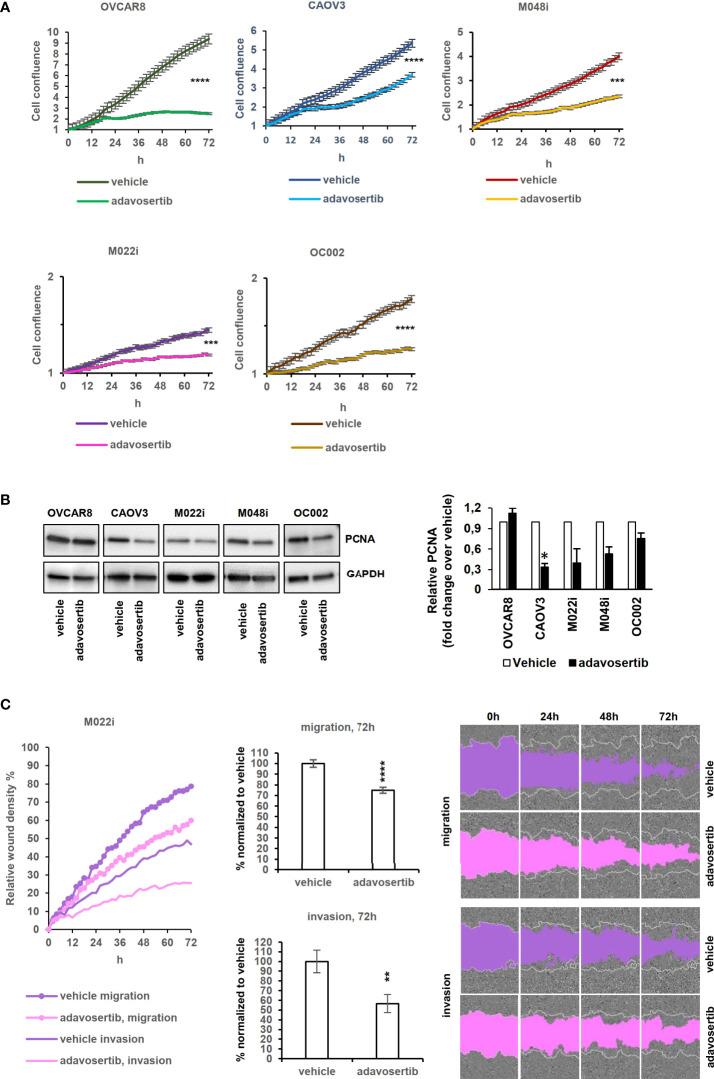
Effects of adavosertib on proliferation and wound healing. **(A)** Proliferation of HGSOC cells treated with adavosertib (500 nM) or vehicle was monitored in IncuCyte every 2 h for 72 h (normalized to time point 0 h). **(B)** Expression of the proliferation marker PCNA (proliferating cell nuclear antigen) protein at 72 (h) The bars show quantitative analysis of PCNA normalized to GAPDH using ImageJ software. The values shown are the mean ± SE of three separate experiments. **(C)** Wound healing experiment of the M022i HGSOC primary cells treated with adavosertib (500 nM) for 72 (h) Data of the four other HGSOC cells (M048i, OC002, OVCAR8, and CAOV3) are presented in Figure S8. Significant difference between vehicle and adavosertib treatment was determined by the *t*-test at ns = p > 0.05, *p ≤ 0.05,**p ≤ 0.01, ***p ≤ 0.001, and ****p ≤ 0.0001).

Proliferation was studied by measuring the confluence area of cell cultures at 2-h intervals. Adavosertib significantly reduced proliferation in all the tested cells (*P* < 0.0001; *t*-test) (**Figure 4A**). Although two of the patient-derived cell lines (M022i and OC002) proliferated very slowly, a clear (58.1% and 67.0%) inhibition of proliferation was observed after 72 h of treatment. For the faster proliferating M048i, CAOV3, and OVCAR8, the reduction was 55.2%, 38.0%, and 82.3%, respectively. Here, again we were not able to show any difference in proliferation between the adavosertib-resistant cell model M048i as compared to the more sensitive HGSOC lines.

The expression of the proliferation marker PCNA (proliferating cell nuclear antigen) was detected with Western blotting to confirm the confluence area-based proliferation result. The amount of PCNA decreased after 72 h of adavosertib treatment in the tested HGSOC lines (**Figure 4B**), except for OVCAR8 in which PCNA remained unchanged.

Wee1 inhibition reduced the mobility of all HGSOC lines as evaluated by both migration and invasion assays (**Figure 4C** and **Figure S12**), with migration showing a greater reduction than invasion through the extracellular matrix.

### Adavosertib induces DNA damage regardless of HR status

**Figure 5:**
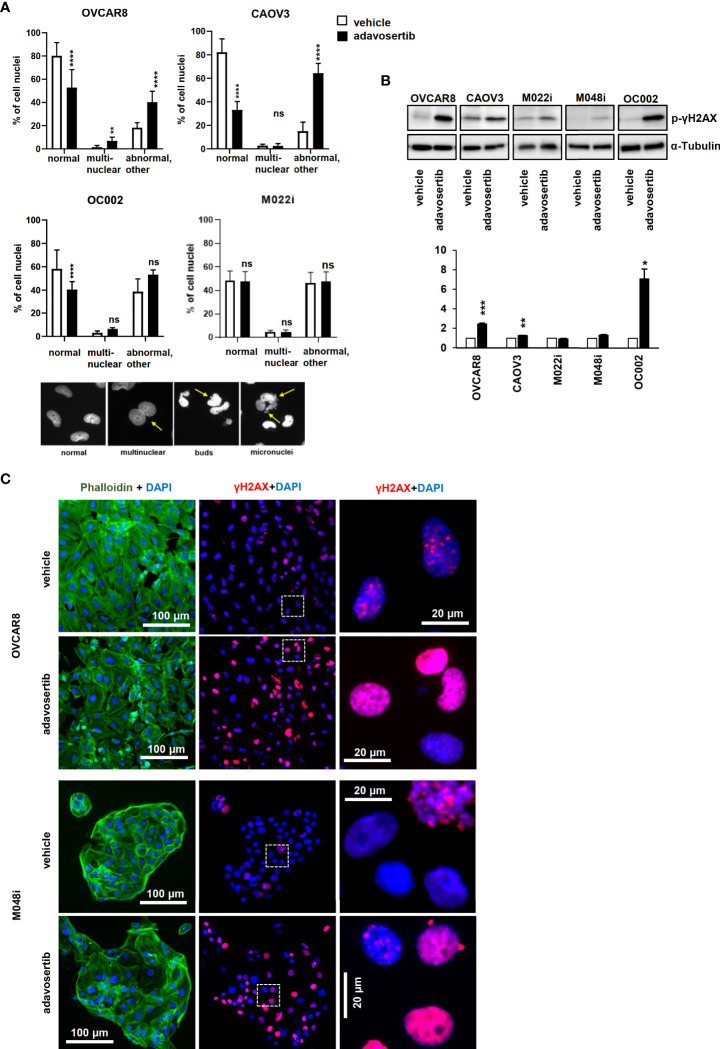
Adavosertib induces nuclear abnormalities and DNA damage **(A)** Nuclear morphology of cells treated with adavosertib or vehicle. Percentage of normal, multinucleated, and abnormal nuclei (micronucleus and buds of OVCAR8, CAOV3, OC002, and M022i cell cultures). Examples of nuclear abnormalities present in the samples: first picture of cells with normal nuclei, the second of a multinuclear cell, the third with a cell with nuclear buds, and the fourth of a cell with several micronuclei caused by the adavosertib treatment. **(B)** Protein levels of DNA-damage marker γH2AX (phosphor S-139) after 72 h of treatment with adavosertib (500 nM). The bars show quantitative analysis of PCNA normalized to α-tubulin using ImageJ software. The values shown are the mean ± SE of three separate experiments. Significant difference between vehicle and adavosertib treatment was determined by the *t*-test at ns = p > 0.05, *p ≤ 0.05,**p ≤ 0.01, ***p ≤ 0.001, and ****p ≤ 0.0001). **(C)** OVCAR8 and M048i cells were cultured on coverslips and treated with 500 nM adavosertib for 72h. Cells were fixed and stained with γH2AX (phosphor S-139) for DNA damage, DAPI for cell nuclei, and phalloidin for the actin filament (green, actin; blue, nucleus; and red, DNA damage).

The impact of Wee1 inhibition on DNA damage and nuclear morphology was further addressed. Apart from M022i, adavosertib induced a significant increase in the number of aberrant nuclei (multinucleated, bud, and micronuclei) in the HGSOC lines (**Figure 5A**). Similarly, using both Western and immunofluorescence imaging, the DNA-damage marker γH2AX (phospho S-139) was clearly upregulated in all the tested cells (**Figures 5B, C**).

The efficacy of many current HGSOC drugs depends on the homologous recombination DNA repair capacity of the cancer cells. Therefore, we wanted to evaluate whether this is also the case for adavosertib. The HGSOC lines were evaluated for homologous recombination (HR) capacity using both genomic (**Table S2**) and functional (**Figure S13** and **Table S14**) tests. Of the cell lines, OC002 and OVCAR8 were HR-deficient (HRD) while M022i, M048i, and CAOV3 were HR-proficient (HRP) (**Table S2**, **Figure S13**, and **Table S14**). The result suggests that the effects of adavosertib are independent of the HR status of the cells.

## Discussion

Despite the advances in understanding the molecular background of HGSOC, patients who do not initially respond or acquire resistance to platinum compounds or PARP inhibitors have limited treatment options. Based on the high-throughput screen of HGSOC cell lines, and with a focus on compounds in the clinical development, we identified two compound groups cytotoxic to HGSOC independent of the cell culture method: Wee1 inhibitor (adavosertib, AZD1775) and Hsp90 inhibitors (BIIB021, alvespimycin, luminespib, and tanespimycin). In a further validation using three patient-derived and two conventionally available HGSOC cell lines, adavosertib provided the best cytotoxicity result. Wee1 has already proven to be a potential target in genomically unstable cancers, including HGSOC, due to its role in cell-cycle control and DNA-damage response (DDR) pathways [reviewed in (25–28)]. Our findings presented here indicate that adavosertib inhibits HGSOC cell growth at multiple levels. Importantly, the effect in our study material is independent of the homologous recombination capacity of the cells and thus potentially effective in patients who do not benefit from current treatments.

We also noticed generally similar effects on the three HRP and two HRD cell lines. Additionally, adavosertib inhibited equally well patient-derived HGSOC cells and the publicly available HGSOC cell lines. One of the patient-derived HGSOC cell lines, M048i, was less responsive than the other cell lines in the high-throughput cytotoxicity assay to most of the 306 drugs including adavosertib for reasons that remain unclear. No significant difference was found in the DNA sequencing or RNA expression data of the multidrug resistance (MDR) ABC transporters (ATP-binding cassette transporters) compared to other cell lines (**Figure S15**). ABC transporters are known to play a role in the MDR mechanism in cancer, and they are responsible for the increased efflux rate of anticancer drugs in the MDR phenomena (29, 30). In spite of the relative resistance in the conventional cytotoxic assay, M048i responded to adavosertib in other assays, indicating that none of the investigated cell lines were unresponsive to Wee1 inhibition.

Our study supports several mechanisms of action for adavosertib. Mechanistically, Wee1 regulates the G2/M checkpoint *via* inhibiting CDK1 and delays the mitosis entry of cells with DNA damage. Its inhibition in cells with DNA damage allows these cells to enter mitosis prematurely, which leads to mitotic catastrophe and apoptosis. Wee1 is also involved in regulating the G1/S checkpoint *via* CDK2 and thereby DNA replication by phosphorylating CDK2-bound cyclin A/E during the S phase (31, 32). The exact mechanisms of Wee1 inhibition in addition to the G2/M checkpoint is not yet completely understood. Heijink et al. (2015) found in their study that Wee1 inhibitor sensitivity is controlled by the status of several S-phase entry genes including CDK2 (33). In line with our results, they correspondingly report elevated γH2AX after Wee1 inhibition. We also found that adavosertib induced increased levels of the γH2AX DNA damage marker and shortened the S phase together with the abnormalities in the nuclei, which are indicators of increased replication stress. We found a reduction in CHK1 and an increase in γH2AX following adavosertib therapy, which was consistent with two earlier investigations using breast cancer, pancreatic, and osteosarcoma cell lines (34, 35).

In our study, Wee1 inhibition effectively reduced proliferation and increased apoptosis of all the tested cell lines including the less sensitive patient-derived cell line M048i. An exception was the OVCAR8 cell line that had no change in the PCNA levels compared to the other cells. PCNA plays an important role in replication and interacts with the cell-cycle progression machinery (36). We observed an accumulation of cells in the G2/M phases and a decrease in the S phase after adavosertib treatment. This is also in line with Heijink et al.'s study where they observed a shortened S phase (33). In addition, a similar increase in the G2/M phase has been shown in OVCAR8 cells in a recent study (37). A decreased cyclin B1 expression in all cell lines except in OVCAR8 might indicate the cytotoxic effect to be in the S phase rather than at the G2/M checkpoint. In a normal cell cycle, cyclin B expression peaks in the late G2 phase and radically declines in mitosis (38). The unchanged levels of PCNA and cyclin B1 expression in OVCAR8 cells might indicate that the cytotoxic effect in this cell line lies more in the mitotic catastrophe than in the S phase, compared to the other cells investigated.

Our study demonstrates that adavosertib treatment impairs migration and invasion in HGSOC cell lines, which could in part be interpreted as a consequence of cell-cycle arrest. Other studies have shown similar results in gastric cancer cell lines after Wee1 siRNA-mediated knockdown (39) or adavosertib treatment, although the mechanism of action was not elucidated (40). More recently, Bi et al. (2019) described that targeting Wee1 by shRNA or adavosertib significantly diminished the migration and invasion in esophageal squamous cell carcinoma by suppression of metalloproteinases MMP-2 and MMP-9 (41).

Several clinical trials have shown encouraging cytotoxic efficacy by adavosertib both as a single agent treatment and as a combination therapy in several solid tumors (11–14, 42–45). Thus, a combination therapy approach with platinum compounds or PARP inhibitors has been taken into clinical trials (NCT01357161, NCT03579316, NCT03345784, NCT02272790) (12–14). However, patients that are HRP and platinum resistant do not benefit from these drug combinations. Several other drug combinations have been studied in both *in vitro* and clinical trials. A recent clinical trial reported promising results with adavosertib in combination with nucleoside analog gemcitabine in treatment of platinum-resistant ovarian cancer (14). In an *in vitro* study, a similar result was reported that suggested that Wee1 inhibition sensitizes cells to gemcitabine but also reduced the ATR/CHK1 activity (35).

According to our knowledge, a thorough validation and mechanistic evaluation of Wee1 inhibition mechanisms in patient-derived HGSOC cells has been lacking. The current findings and our previous results showing that adavosertib is also effective against HGSOC cells with stemness features and had an observed cytotoxic effect in all of the studied HGSOC lines including the generally quite resistant M048i cells have increased our understanding of its broad mechanisms of action during the cell cycle.

Importantly, we observed that adavosertib inhibits HGSOC cells regardless of their HR status. A study by Garcia and others (2017) found that adavosertib impairs HR and may work as a combination therapy with PARP inhibitor olaparib in BRCA1/2-mutant leukemias (46). Another study with HRD and HRP murine cell lines demonstrated that Wee1 inhibitor in combination with olaparib had no difference in effectiveness between the HR-deficient and -proficient cells (47). Both of these studies were performed in combination with PARP inhibitor olaparib. We were able to demonstrate that adavosertib has efficacy toward HRP cells also as a single agent.

Our findings lay the basis for treatment of HRP patients, with whom treatment strategies are scarce. It is especially important to find an effective DNA-damaging agent to be used in combination with adavosertib for the platinum-resistant HGSOC patients. In this regard, gemcitabine has already shown promising results.

In conclusion, our study suggests Wee1 inhibitor adavosertib as a candidate compound to treat HGSOC patients independent of the HR status of the tumor.

## Data availability statement

The raw sequencing data are available from the European Genome-Phenome Archive (EGA). RNA data is under accession number EGAD00001006456 and DNA data is available under same DAC (EGAC00001001760).

## Ethics statement

The studies involving human participants were reviewed and approved by Ethics Committee of the Hospital District of Southwest Finland (ETMK): ETMK 53/180/2009 §238 and ETMK 69/180/2010 and The Finnish National Supervisory Authority for Welfare and Health in Finland (Valvira): DNRO 6550/05.01.06/2010 and STH507A.The patients/participants provided their written informed consent to participate in this study.

## Author contributions

PR, AA, and OC conceived the project. JH, SG, and AA contributed to the material acquisition. KK and PR established and maintained patient-derived HGSOC cell lines. PR, AS, VH, SaP, and KK designed the experiments. PR, AS, VH, PM, SaP, and SwP carried out the experiments. PR, AS, VH, KH, SaP, YL, and JO analyzed the data. PR, AS, and VH generated the figures. PR prepared the original draft. All authors reviewed and commented on the article. All authors contributed to the article and approved the submitted version.

## Funding

The project has received funding from EU Horizon 2020 (667403), The Academy of Finland (292606), Finnish Cancer Society, Sigrid Juselius Foundation, Finska Läkaresällskapet, and Helsinki University Hospital Research Funds. PR has received research grants from K. Albin Johanssons Stiftelse, Varsinais-Suomen rahasto (85211878), Orion Research Foundation, Lounais-Suomen Syöpäyhdistys, University of Turku Postgraduation Education Unit, and Ida Montinin Säätiö.

## Acknowledgments

We thank the DDCB core facility (FIMM HTB unit) supported by the University of Helsinki and Biocenter Finland. We also thank CSC – IT Center for Science Ltd. for computational resources.

## Conflict of interest

AA reports receiving honoraria and travel support from GSK and is a member in Nordic Society of Gynecologic Oncology NSGO-CTU Foundation Board. KW reports receiving payment for attending and presenting at meetings organized by Pfizer and Amgen. JH has received funding from EU Horizon 2020 (965193).

The remaining authors declare that the research was conducted in the absence of any commercial or financial relationships that could be construed as a potential conflict of interest.

## Publisher’s note

All claims expressed in this article are solely those of the authors and do not necessarily represent those of their affiliated organizations, or those of the publisher, the editors and the reviewers. Any product that may be evaluated in this article, or claim that may be made by its manufacturer, is not guaranteed or endorsed by the publisher.
