# Supplementary figures and images for: Effects of Wee1 inhibitor adavosertib on patient-derived high-grade serous ovarian cancer cells are multiple and independent of homologous recombination status
Source: Front Oncol. 2022 Aug 23;12:954430. doi: 10.3389/fonc.2022.954430 (PMC9445195; doi:10.3389/fonc.2022.954430)

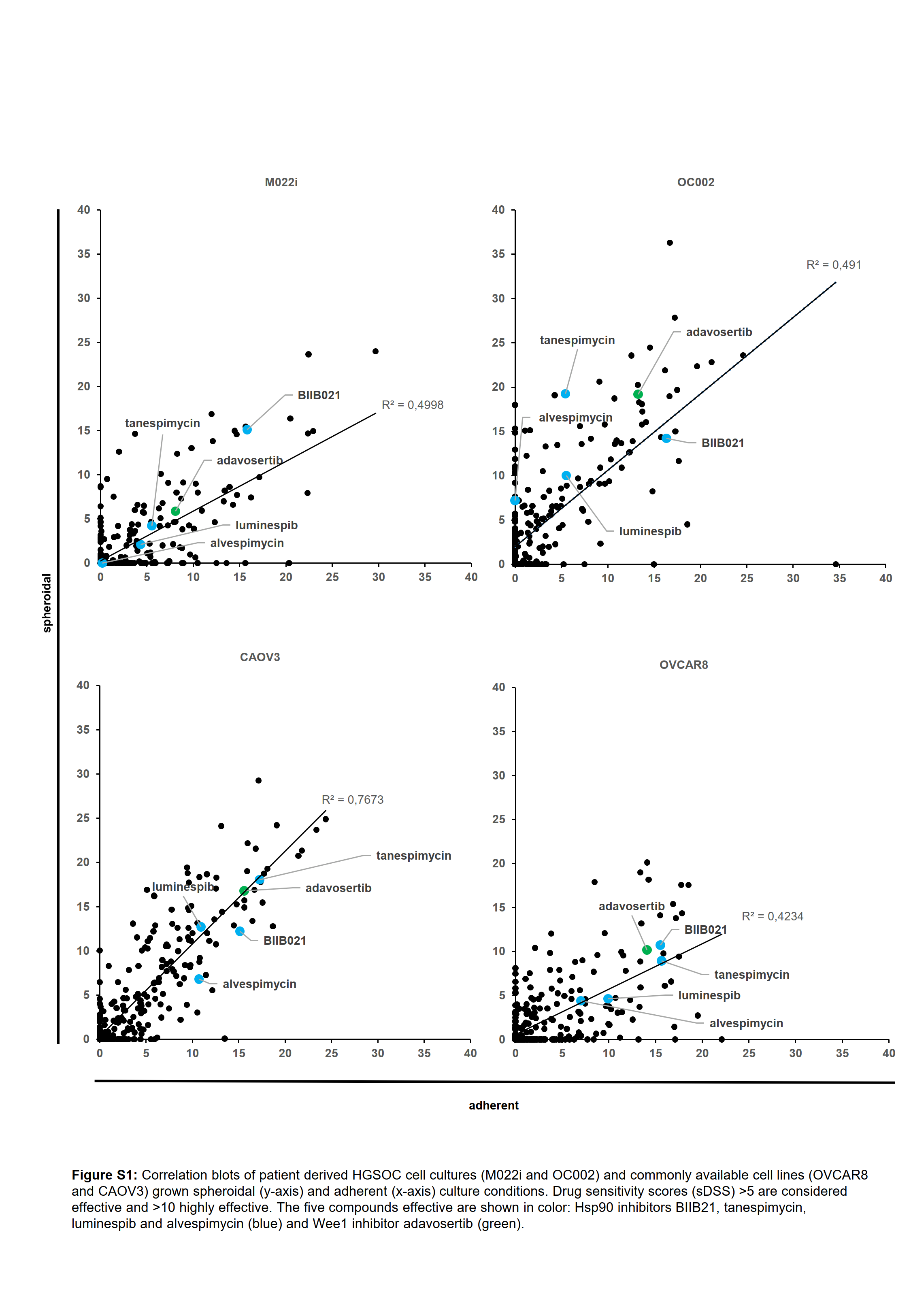

Supplement: Supplementary file 1 [file Image_1.tif]

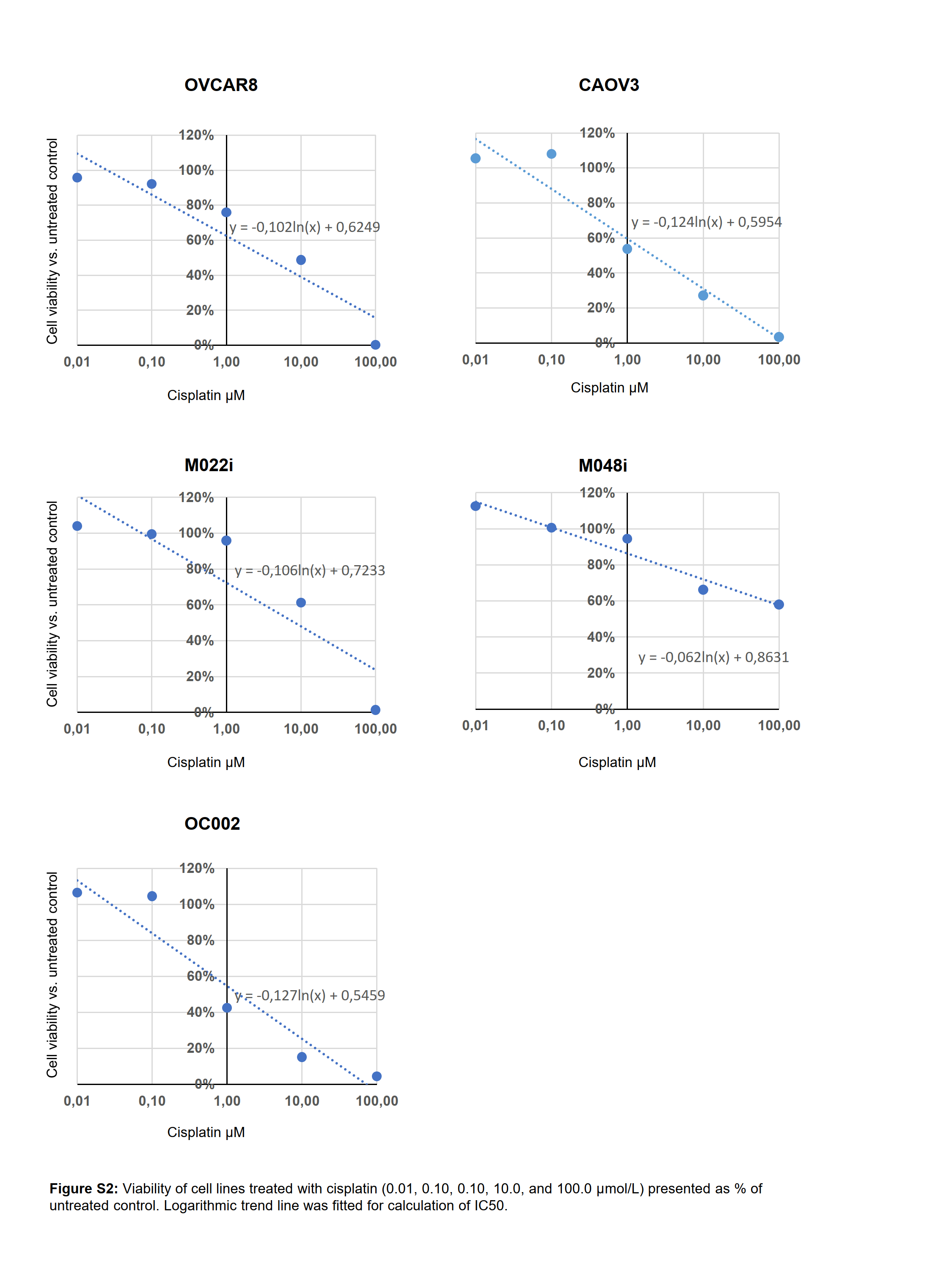

Supplement: Supplementary file 2 [file Image_2.tif]

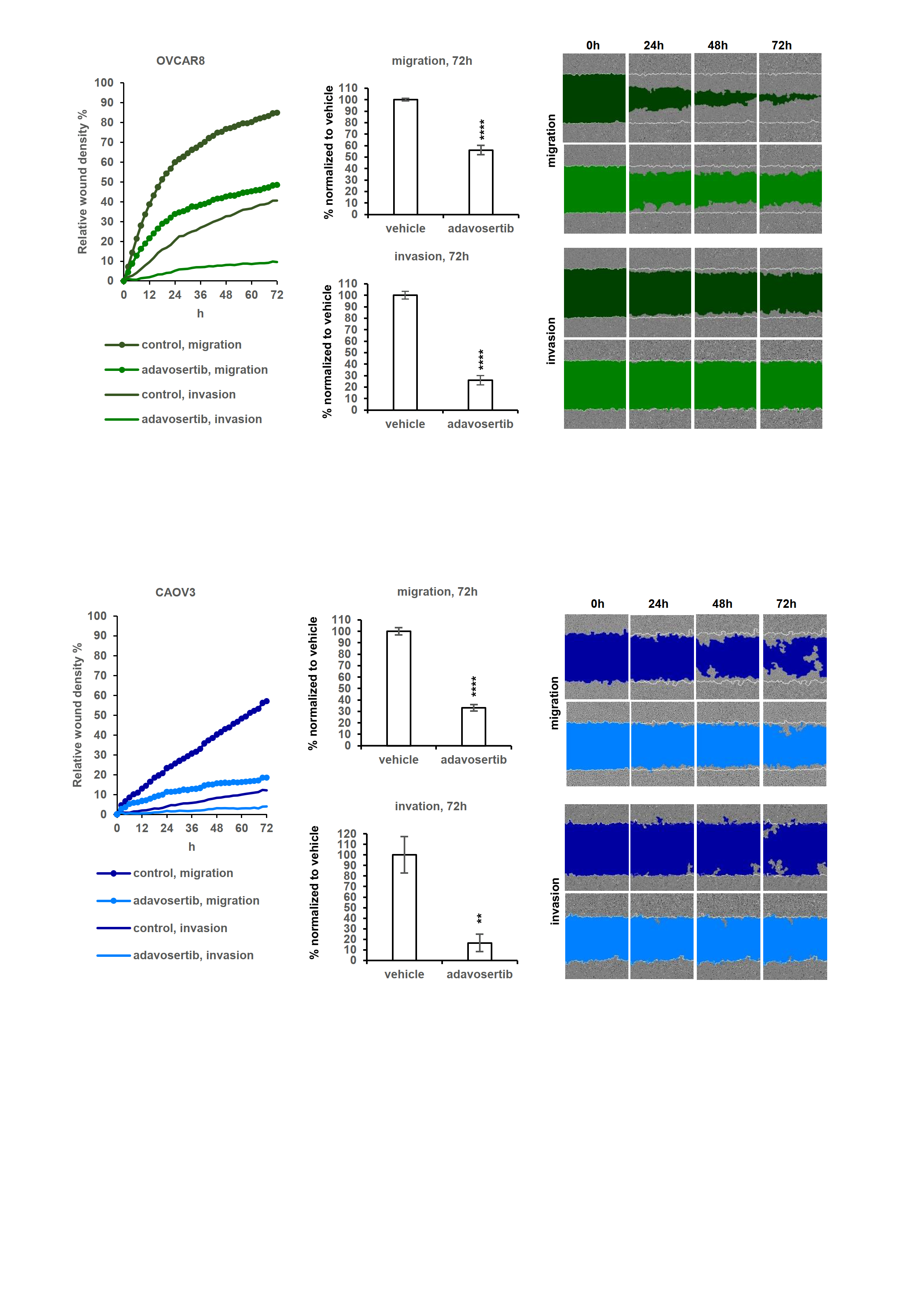

Supplement: Supplementary file 3 [file Image_3.tif]

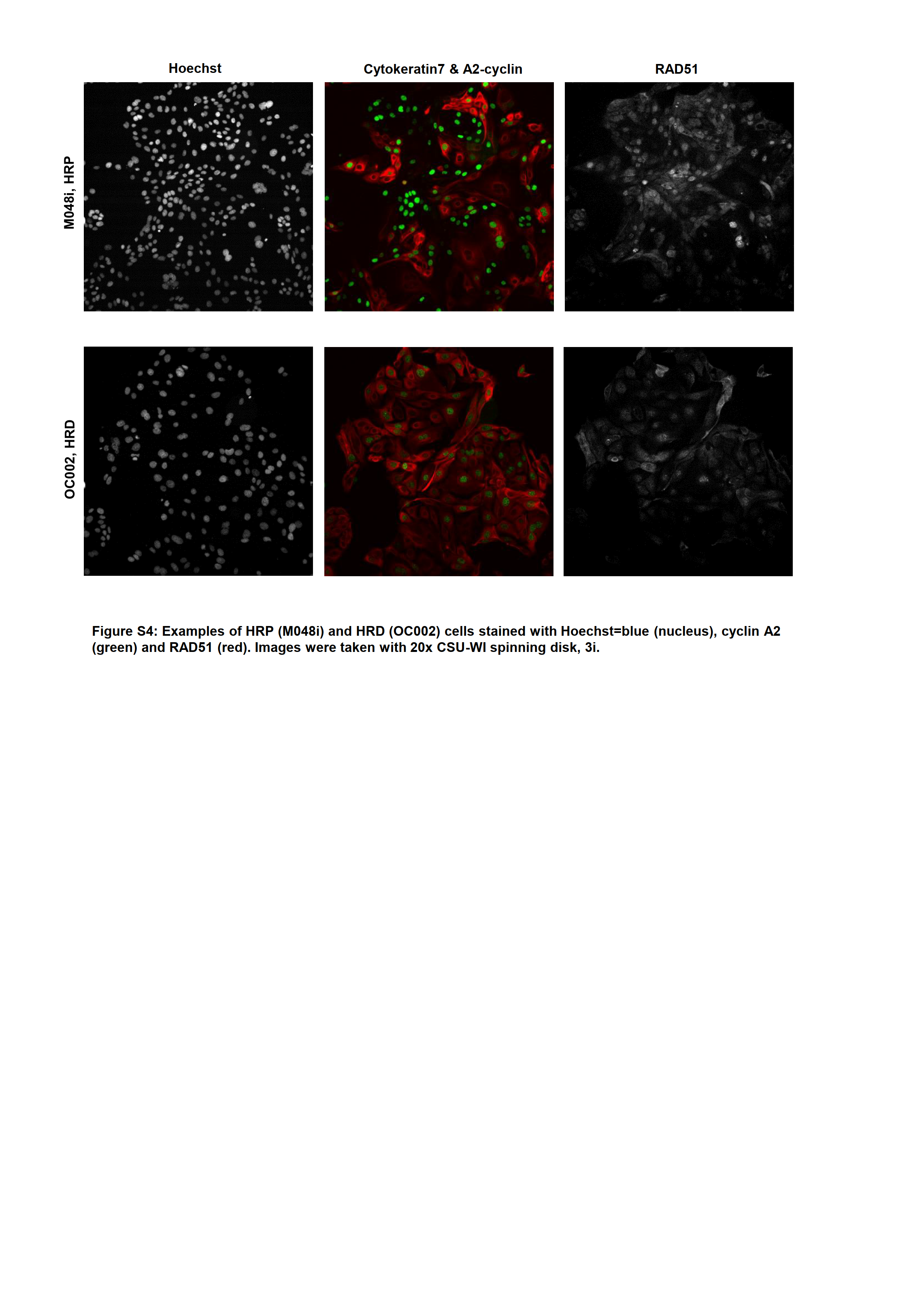

Supplement: Supplementary file 4 [file Image_4.tif]

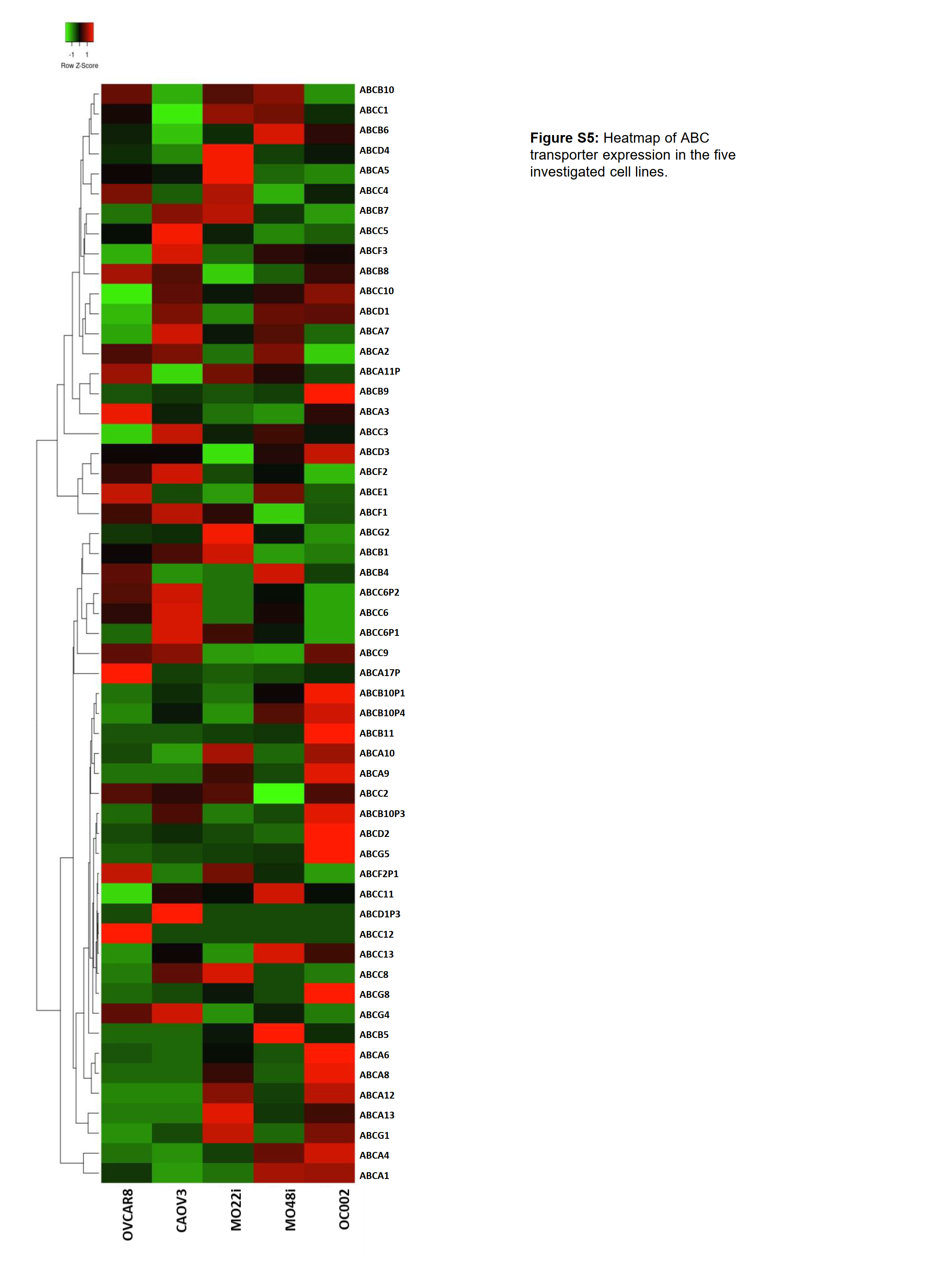

Supplement: Supplementary file 5 [file Image_5.tif]
